# Bear in mind: the role of personal background in semantic animal fluency – The SMART-MR study

**DOI:** 10.3389/fpsyg.2023.1227053

**Published:** 2023-09-22

**Authors:** Annelot P. Smit, Magdalena Beran, Emma L. Twait, Mirjam I. Geerlings, Jet M. J. Vonk

**Affiliations:** ^1^Julius Center for Health Sciences and Primary Care, University Medical Center Utrecht and Utrecht University, Utrecht, Netherlands; ^2^Amsterdam UMC, Department of General Practice, Vrije Universiteit, Amsterdam, Netherlands; ^3^Research Institute Amsterdam Public Health, Research Programme Aging and Later Life, and Research Programme Personalized Medicine, Amsterdam, Netherlands; ^4^Research Institute Amsterdam Neuroscience, Research Programme Neurodegeneration, and Research Programme Mood, Anxiety, Psychosis, Stress, and Sleep, Amsterdam, Netherlands; ^5^Amsterdam UMC, Department of General Practice, University of Amsterdam, Amsterdam, Netherlands; ^6^Department of Neurology, Memory and Aging Center, University of California, San Francisco (UCSF), San Francisco, CA, United States

**Keywords:** cognition, category fluency, verbal fluency, demographic, personal background

## Abstract

**Objectives:**

Semantic fluency is a prominent neuropsychological task, typically administered within the category ‘animals’. With the increasing development of novel item-level metrics of semantic fluency, a concern around the validity of item-level analyses could be that personal background factors (e.g., hobbies like birdwatching or fishing) may disproportionally influence performance. We analyzed animal fluency performance at the item level and investigated the prevalence of individuals with abundant knowledge in specific classes of animals (e.g., birds, fish, insects) and the relationship of such knowledge with personal background factors and other cognitive tasks (episodic memory and executive functioning).

**Method:**

Participants included 736 Dutch middle-aged to older adults from the SMART-MR cohort (mean age 58 ± 9.4 years, 18% women). Individuals were asked to name as many animals as possible for 2 min. Number of people with abundant animal class knowledge was calculated for the ability to recall a series of minimum ≥5 and up to ≥15 animals within a specific class with at most one interruption by an animal from another class. Subsequent analyses to investigate relationships of abundant class knowledge with sociodemographic characteristics (*t*-tests and chi-square tests) and cognitive performance (linear regressions) were performed for a cut-off of ≥10 animals within a specific class (90th percentile), with a sensitivity analysis for ≥7 animals (67th percentile).

**Results:**

A total of 416 (56.2%) participants recalled a series of ≥5 animals from a specific class, 245 (33.3%) participants recalled ≥7, 78 (10.6%) participants recalled ≥10, and 8 (1.1%) participants recalled ≥15. Those who recalled a series of at least 10 animals within a class were older, more often men, and more often retired than those who did not. Moreover, they had a higher total score on animal fluency, letter fluency (i.e., executive functioning), and episodic memory tasks compared to those who did not.

**Discussion:**

Our results suggest that the benefit of abundant animal class knowledge gained by personal background does not disproportionally influence animal fluency performance as individuals with such knowledge also performed better on other cognitive tasks unrelated to abundant knowledge of animal classes.

## Introduction

Semantic knowledge refers to an individual’s capacity to recall ideas, concepts, and facts, including the meaning of words. Semantic knowledge is influenced by language, culture, and sociodemographical factors (Winkler-Rhoades et al., 2010; Eng et al., 2019). Differences in semantic knowledge have been found across age, sex/gender, languages, cultures, and education levels (Tombaugh et al., 1999; Mathuranath et al., 2003; Henry et al., 2004; Mioshi et al., 2006; Van der Elst et al., 2006). These findings are typically demonstrated on a (sub)group level, such as across men versus women; however, differences in semantic knowledge may also be expected at an individual level.

Verbal fluency tests are one of the most commonly used neuropsychological tests to assess semantic knowledge and executive functioning (Lezak and Loring, 2004; Shao et al., 2014). During these tests, participants are asked to name as many words as possible from a given category (e.g., animals) or beginning with a specific letter (e.g., N). Typically, the total number of words generated is used in research and clinical practice as a measure for verbal fluency task performance. However, investigating performance at the item level (i.e., which specific words were generated) can reveal additional insights and may be a more sensitive measure of cognitive functioning. For example, item-level psycholinguistic metrics of semantic fluency related to APOE e4 status—a genetic risk factor of Alzheimer’s disease (AD)—while the traditional total word count did not (Vonk et al., 2019). The development and use of item-level metrics of semantic fluency in aging and dementia research has flourished in recent years (Rofes et al., 2020; Taler et al., 2020; Saranpää et al., 2022; van den Berg et al., 2022).

Multiple studies showed that personal background, such as an individual’s acquired knowledge based on semantic and episodic memory through life experiences, plays an important role in recalling words from a specific category. For instance, on a semantic fluency task for the category “cars,” Walker and Kintsch (1985) showed that participants were more likely to recall cars they owned. Personal background factors, including demographics, are often considered as indicators of “cognitive reserve”, which is a capacity build by life-time experiences to maintain cognitive function despite the presence of neurodegeneration (Stern et al., 2020). These lifetime experiences can for example be related to education, occupation, and leisure activities. As such, it may be probable that the ability to recall animals is influenced at an individual level by one’s hobbies or profession. Someone who enjoys birdwatching or fishing, with abundant knowledge in these animal classes, may be more likely to recall more animals from these classes than one that does not have such hobbies.

This study examined the role of personal background in animal fluency performance, under the assumption that individuals who gained abundant knowledge about animal classes in their life will likely recall them more often on an animal fluency task (following Walker and Kintsch, 1985). We investigated the prevalence of individuals with abundant animal class knowledge (i.e., listing a series of animals within a specific class, e.g., birds, fish, insects) in semantic animal fluency in our sample of cognitively normal middle-aged to older adults. Subsequently, we investigated if these individuals who showed abundant animal class knowledge differed on personal background characteristics from those who did not. Based on the literature, we hypothesized that those who showed abundant animal class knowledge would be more often men, older, and more often retired than those who did not (Schroeder et al., 2006; Agahi and Parker, 2008; Moore et al., 2008; Gibson and Singleton, 2012). To examine the concern around the validity of item-level analyses that personal background may disproportionally benefit animal fluency performance relative to other cognitive tasks, we also analyzed if the groups differed in performance on the cognitive domains of episodic memory and executive functioning.

## Methods

### Study population

Participants were drawn from the second visit of the Second Manifestations of ARTerial disease-Magnetic Resonance (SMART-MR) study. Recruitment and detailed procedures in SMART-MR have been described elsewhere (Geerlings et al., 2010). Between 2001 and 2005, 1,309 participants with a history of coronary artery disease, cerebrovascular disease, peripheral arterial disease, or abdominal aortic aneurysm registered at the University Medical Center (UMC) Utrecht were enrolled (Geerlings et al., 2009, 2010). We used data from their follow-up visit approximately 4 years later (*n* = 754; retention rate visit 1 to 2 = 57.6%), in which an extended cognitive battery with semantic fluency was administered. Participants were invited for a one-day visit at the UMC that included neuropsychological tests, a 3-dimensional T1-weighted MR image, and blood and saliva samples. Information on demographics, risk factors, and depressive symptoms were assessed via questionnaires. Written informed consent was received from all participants, and the SMART-MR study was approved by the ethics committee of the UMC. The current study was a secondary analysis of the data collected in the SMART-MR cohort.

### Animal fluency data

Assessment of animal fluency was administered to SMART-MR participants as of their second visit. During the animal fluency task, participants were asked to name as many animals as possible within 2 min. The answers were written down on paper by the interviewer. Some participants recalled animals so rapidly that the interviewer could not always keep up and record the specific animal, and these animals were instead recorded on paper as a plus sign (+). Animals were marked as incorrect when they were recalled more than once (i.e., repetitions), in a different language than Dutch, in plural forms after naming the singular form, or as a name of a popular animal (e.g., Willy from Free Willy) or their pet’s name (e.g., Buddy). The specific words generated during the animal fluency task were entered into a database. For the second SMART-MR visit, item-level animal fluency data was available for 736 individuals (97.6% of total N).

To categorize animal classes, we used the animal cluster scheme from the Semantic Network and Fluency Utility (SNAFU) (Zemla et al., 2020), which is based on schemes by Troyer et al. (1997) and Hills et al. (2012). We adapted this scheme by translating it into Dutch and further expanding the categorization to include all the animals generated by our sample. Animal classes are a subordinate of clusters, since animal classes are typically clusters, but not all clusters are animal classes; for example, “pets” or “zoo animals” are typical clusters, but are not considered animal classes. We considered abundant knowledge in the following animal classes: “Bird,” “Bovine,” “Canine,” “Deers,” “Feline,” “Fish,” “Insects,” “Primates,” “Reptile/Amphibian,” “Rodents,” “Weasels,” and “Worms.”

Among the 736 animal fluency tasks, data of 70 participants (9.5%) included at least one plus sign. Among those, data of 40 participants (5.4% of 736) included more than one plus sign. Among those data that included more than one plus sign per participant, 3 (0.4% of 736) had more than one plus sign in a row while producing animals within a specific class. These calculations highlight that if an interviewer was not able to record the specific animal that was generated by the participant, it was typically an isolated event. As such, if a participant’s output contained one or more plus signs in a row and recalled animals from the same class before and after the plus sign(s), we assumed the missing values were likely animals from the same class. Even if the animal were to be from a different class, this method still lines up with our policy to allow at most one interruption by an animal from another class (see section “Definition of abundant animal class knowledge”). To ensure this method was adequate, we performed additional analyses (not reported) in which we deleted participants with more than 20% plus signs in their performance (*n* = 31), which did not change the associations between showing abundant animal class knowledge and the personal background factors.

### Definition of abundant animal class knowledge

Due to the lack of an existing definition for abundant animal class knowledge on an animal fluency task, we created an *ad-hoc* definition based on the following considerations. We postulated that someone is knowledgeable in an animal class if they are able to recall a consecutive series of exemplars within one animal class that is at most interrupted by one animal from another class. While we adhere to the practice of consecutiveness following classical clustering analysis, we allowed an interruption in series to capture semantic structure more appropriately than in classic clustering analysis, since the latter is sometimes considered a metric of executive control (Fong et al., 2020; Vonk et al., 2023).

We reasoned that a relatively small proportion of the participants (~10–20%) should be labeled as having abundant animal class knowledge compared to their peers, based on the conception that scoring above the 80–90th percentile on a test is generally considered high performance. We explored abundant knowledge of an animal class by calculating the number of participants that could name between 5 and 15 animals consecutively within an animal class with at most one interruption by an animal from another class. If someone showed abundant knowledge in more than one animal class (e.g., they recalled eight insects and also nine birds), the longest series was selected to define the specific class of the person’s abundant animal class knowledge.

Based on these results (described in detail in the Results section), we decided to analyze individuals who could generate a series of at least 10 exemplars of a class to comply with the percentile conception (i.e., 90th percentile, putting these individuals in the top 10% of animal class knowledge). To test robustness of results and to increase statistical power, which is based on the smallest group’s sample size, we also included a second (sensitivity) analysis of individuals who could generate a series of at least seven exemplars of a class (67th percentile; approximately one standard deviation from the mean, which is typically defined as above average).

### Personal background factors

Age, sex/gender, and education level were self-reported measures. As it was unknown whether an individual reported their biological sex or their gender identification, we refer to this variable as “sex/gender”. Education levels were based on the Dutch school system, ranging from no primary school to an academic degree; we divided these eight groups into low (less than a high school education), medium (at least some high school education), and high (college or university) education categories.

Information on participants’ profession was administered as one of 10 pre-defined categories: executive profession, senior administrative profession, technical and related professions, administrative and sales professions, craft professions, uneducated staff, self-employed, military profession, multiple professions, or other. Participants with an executive profession included those with jobs such as physician, lawyer, or architect. Professions such as employees of private companies, government and civil service or government officials were assigned the senior administrative profession category. Technical and related professions were professions such as non-academic engineer, IT, safety and quality assurance specialist, lab technician, or nurse. Administrative and sales professions included jobs such as shop staff, receptionists, secretaries, service staff, or specialist drivers. Craft professions were participants with a profession as a plumber, carpenter, mechanic, or electrician. Factory workers, cleaning staff, or unskilled agricultural staff were assigned to uneducated staff. Entrepreneurs, freelancers, writers, musicians were assigned to the self-employed profession. Due to small numbers in the categories of military profession, multiple professions, and other profession, these categories were not included in the analyses comparing the distribution of professions between groups. Similarly, small numbers in the uneducated staff profession led to excluding this category in the main analysis, but was included in the sensitivity analysis (≥7 animals within one class).

Participants were asked by use of a questionnaire if they held a paid job at the moment of their visit. This question could be answered with “Yes,” “No, I’m looking for a job,” “No, I’m houseman/wife,” “No, I’m retired,” “No, I’m incapacitated,” or “Other.”

### Cognitive functioning

While the animal fluency task is typically considered to reflect semantic processing with a component of executive functioning, the letter fluency task is typically considered mainly a task of executive functioning (i.e., updating, inhibition, and lexical access) (Shao et al., 2014; Vonk et al., 2019). The Mini-Mental State Examination (MMSE) was used to reflect overall global cognitive functioning of the population sample (Folstein et al., 1975). We used a cut-off score of >26 to define those who are cognitively unimpaired. Verbal episodic memory was assessed using the total number of words recalled during the immediate recall and delayed recall of the 15 Word Learning Test (15-WLT) (Brand and Jolles, 1985). Visual episodic memory was assessed using the participant’s score on the delayed recall of the Rey-Osterrieth Complex Figure test (Osterrieth, 1944). For these tasks, higher scores reflected higher levels of memory functioning. Lastly, to have an account of vocabulary level we included the Dutch Adult Reading Test [DART (Schmand et al., 1998); Dutch version of the National Adult Reading Test, i.e., NART], which is a vocabulary measure based on reading recognition of irregularly spelled words.

### Statistical analyses

Participants in SMART-MR with missing data on the animal fluency task were excluded (*n* = 18). We calculated the prevalence in our sample of having abundant animal class knowledge as the percentage of individuals that recalled a series of *X* or more animals from a specific class, with *X* ranging from 5 to 15 exemplars. We also evaluated the frequency distributions of which specific animal classes were recalled in a series.

Differences in personal background factors between those who showed abundant animal class knowledge by generating a series of ≥10 animals within a class (90th percentile) and those who did not were investigated using *t*-tests for continuous and chi-square tests for categorical variables. Regarding profession, “Military profession,” “Multiple profession,” and “Other” were removed from the chi-square test because of the low number of participants executing these professions (respectively 11, 20, and 5 participants). Due to a small number of responses in most categories on the question if they held a paid job (all below ≤64 participants), except for “Yes” and “No, I’m retired,” we could only compare groups on these two categories. From now on, we will therefore refer to this variable as “working status.” As a sensitivity analysis, we also ran these analyses with individuals that generated ≥7 exemplars (67th percentile, approximately one standard deviation from the mean) from the same animal class in a series with at most one different animal in between.

Subsequently, we investigated the relationship between showing abundant animal class knowledge (independent variable) and episodic memory and executive functioning (dependent variable in separate models) with linear regressions adjusted for age, sex/gender, and education (covariates).

Multiple comparisons were corrected for with a False Discovery Rate (FDR) approach using the Benjamini–Hochberg procedure (Benjamini and Hochberg, 1995). In short, value of ps of the effect of interest were ordered from smallest to largest and ranked *i* = 1 through *i* = 6, respectively. The Benjamini–Hochberg critical value was calculated as (*i*/*m*)*Q* where i is the rank, m is the total number of tests within the same set of statistical inferences (i.e., 6), and *Q* is the false discovery rate [set at 0.10; see Vonk et al. (2019)]. The largest *p*-value in the ranked order that is smaller than the critical value plus all *p*-values preceding it in rank are considered significant.

All analyses were performed in R (version 3.6.1) and all code is available at: github.com/jmjvonk.

## Results

### Prevalence of abundant animal class knowledge

**Figure 1:**
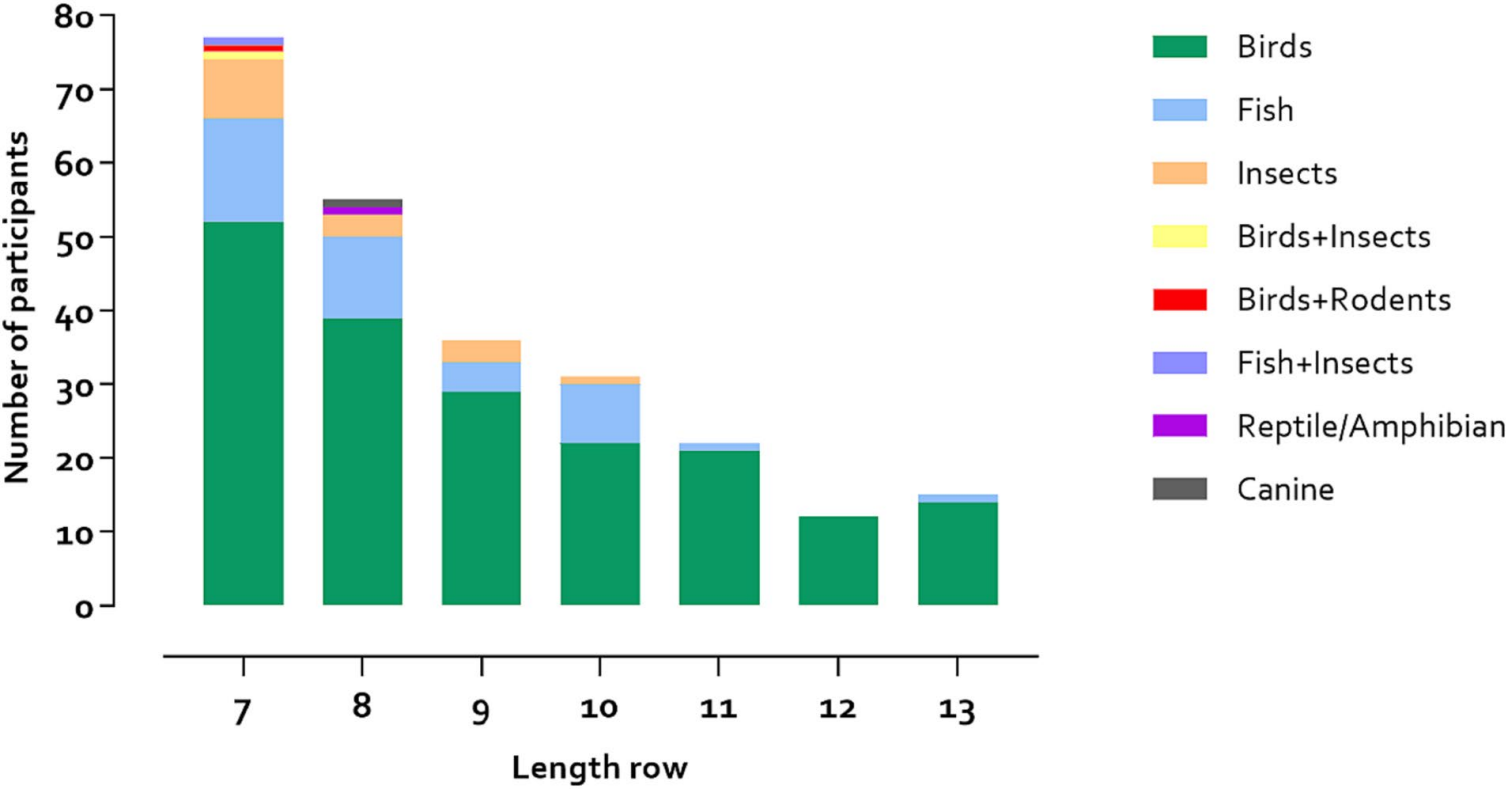
Distributions of animal classes among those that recalled 7–13 animals from a specific class.

**Figure 2:**
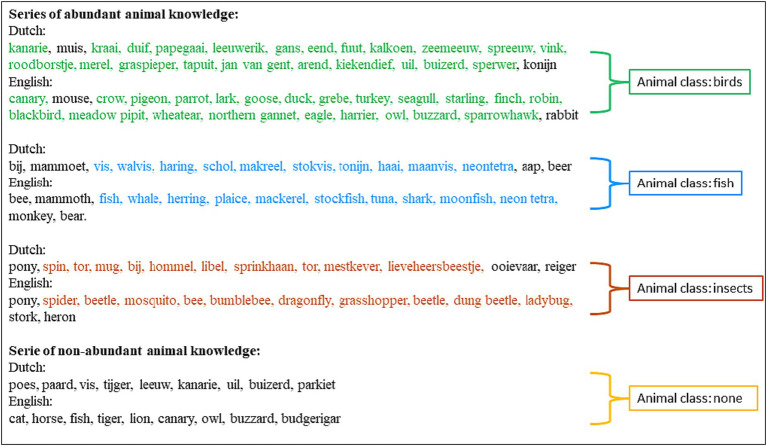
Examples of series of (non-)abundant knowledge of animal class.

Based on our sample of 736 participants, Table 1 shows the number and percentage of participants who recalled 5 to ≥15 animals from a specific class consecutively with at most one interruption. A cut-off score of ≥7 animals recalled from a specific animal class resulted in 33.3% of the sample labeled as having abundant animal class knowledge, and a cut-off score of ≥10 animals reflected 10.6% of the total sample. The frequency distribution of which specific classes were recalled are displayed in Figure 1. The variety in animal classes decreased with increasing number of animals recalled in a series; particularly birds and fish tended to be recalled in longer series (examples of series in Figure 2).

**Table 1:** Overview number and prevalence of participants per X recalled animals from a specific class consecutively (with at most one interruption).

Animal class series recalled	Number of participants	Prevalence (%) in sample (N = 736)
≥5	416	56.2
≥6	331	45.0
≥7	245	33.3
≥8	169	23.0
≥9	114	15.5
≥10	78	10.6
≥11	49	6.7
≥12	27	3.7
≥13	15	2.0
≥14	11	1.5
≥15	8	1.1

### Influence of personal background factors

Baseline characteristics split by those who recalled ≥10 animals in a series (i.e., abundant animal class knowledge) and those who did not are shown in Table 2. Those who showed abundant animal class knowledge (≥10) were older than those who did not (*t*(101) = −2.2, *p* = 0.03, 95% CI = [−4.41; −0.27]), more often men (*X*^2^ (1, *N* = 736) = 5.3, *p* = 0.02), and more often retired than those who did not (*X*^2^ (1, *N* = 599) = 4.6, *p =* 0.03). No differences in education (*X*^2^ (2, *N* = 736) = 2.3, *p =* 0.32), distribution of professions (*X*^2^ (5, *N* = 555) = 2.8, *p =* 0.74), and MMSE-score (*X*^2^ (1, *N* = 736) = 0.5, *p =* 0.48) were found between groups. These results did not change after FDR correction.

**Table 2:** Baseline characteristics split by abundant animal class knowledge (<10 vs. ≥10).

	Non-abundant knowledge (*n* = 658)	Abundant knowledge (*n* = 78)
Age (mean ± (SD), range [])	57.4 (9.5), [27–79]	59.7 (8.6), [41–76]
Sex/gender (% female)	125 (19.0)	6 (7.7)
*Education*		
Low (%)	61 (9.3)	5 (6.4)
Medium (%)	429 (65.2)	48 (61.5)
High (%)	162 (24.6)	25 (32.1)
Animal fluency score/2 min (mean ± SD)	24.7 (8.6)	30.3 (8.4)
Letter fluency score (mean ± SD)	11.6 (4.6)	12.7 (4.6)
*Profession*		
Executive profession (%)	42 (6.4)	4 (5.1)
Senior administrative (%)	111 (16.9)	12 (15.4)
Technical and related professions (%)	89 (13.5)	12 (15.4)
Administrative and sales professions (%)	85 (12.9)	8 (10.3)
Craft professions (%)	103 (15.8)	17 (21.8)
Uneducated staff (%)	47 (7.1)	1 (1.3)
Self-employed (%)	62 (9.4)	10 (12.8)
Military profession (%)	11 (1.8)	0 (0.0)
Multiple profession (%)	20 (2.9)	0 (0.0)
Other (%)	5 (0.8)	0 (0.0)
*Working status*		
Yes	248 (37.7)	21 (26.9)
No, I’m looking for a job	13 (2.0)	2 (2.6)
No, I’m houseman/wife	38 (5.8)	4 (5.1)
No, I’m retired	285 (43.3)	45 (57.7)
No, I’m incapacitated	61 (9.3)	3 (3.8)
Other	8 (1.2)	0 (0.0)
MMSE (mean ± SD)	28.6 (1.7)	28.6 (1.7)
*MMSE*		
<=26 (%)	48 (7.3)	8 (10.3)
>26 (%)	610 (92.7)	70 (89.7)
*15-WLT*		
Total words (mean ± SD)	28.5 (10.4)	30.3 (10.8)
Delayed recall (mean ± SD)	8.7 (3.3)	9.3 (3.6)
Rey-Osterrieth (mean ± SD)	19.4 (6.4)	20.0 (6.7)
DART vocabulary	48.7 (14.6)	48.5 (16.6)

The results of the sensitivity analyses—abundant knowledge based on a cut-off score of ≥7 animals within class—showed a similar pattern (Table 3). Those who showed abundant animal class knowledge (≥7) were older (*t*(524) = −3.7, *p* < 0.001, 95% CI = [−4.03; −1.23]), more often men (*X*^2^ (1, *N* = 736) = 13.7, *p* = <0.001), and more likely to be retired (*X*^2^ (1, *N* = 599) = 11.8, *p =* <0.001) than those who did not. We did not observe differences in education (*X*^2^ (2, *N* = 730) = 2.3, *p =* 0.31), professions (*X*^2^ (6, *N* = 603) = 9.0, *p =* 0.17), or MMSE-score (*X*^2^ (1, *N* = 736) = 0.1, *p =* 0.80) between these groups. These results did not change after FDR correction.

**Table 3:** Baseline characteristics split by abundant animal class knowledge (<7 vs. ≥7).

	Non-abundant knowledge (*n* = 491)	Abundant knowledge (*n* = 245)	Missing *n* (%)	Total (*N* = 736)
Age (mean ± (SD), range [])	56.8 (9.6), [27–79]	59.4 (8.9), [30–78]	0 (0.0)	57.6 (9.4), [27–79]
Sex/gender (% female)	106 (21.6)	25 (10.2)	0 (0.0)	131 (17.8)
Education			6 (0.8)	
Low (%)	45 (9.2)	21 (8.6)		66 (9.0)
Medium (%)	325 (66.2)	152 (62.0)		477 (64.8)
High (%)	116 (23.6)	71 (29.0)		187 (25.4)
Animal fluency score/2 min (mean ± SD)	23.2 (8.4)	29.4 (7.9)		25.2 (8.7)
Letter fluency score (mean ± SD)	11.4 (4.5)	12.5 (4.6)		11.8 (4.6)
Profession			97 (13.0)	
Executive profession (%)	32 (6.5)	14 (5.7)		46 (6.2)
Senior administrative (%)	79 (16.1)	44 (18.0)		123 (16.7)
Technical and related professions (%)	61 (12.4)	40 (16.3)		101 (13.7)
Administrative and sales professions (%)	68 (13.8)	25 (10.2)		93 (12.6)
Craft professions (%)	81 (16.7)	39 (15.9)		120 (16.4)
Uneducated staff (%)	38 (7.7)	10 (4.1)		48 (6.5)
Self-employed (%)	43 (8.8)	29 (11.8)		72 (9.8)
Military profession (%)	7 (1.6)	4 (1.6)		11 (1.6)
Multiple professions (%)	15 (2.9)	5 (2.0)		20 (2.6)
Other (%)	4 (0.8)	1 (0.4)		5 (0.7)
Working status			5 (0.7)	
Yes (%)	196 (39.9)	73 (29.8)		269 (36.5)
No, I’m looking for a job (%)	12 (2.4)	3 (1.2)		15 (2.0)
No, I’m houseman/wife (%)	29 (5.9)	13 (5.3)		42 (5.7)
No, I’m retired (%)	195 (39.7)	135 (55.1)		330 (44.8)
No, I’m incapacitated (%)	48 (9.8)	16 (6.5)		64 (8.7)
Other (%)	6 (1.2)	5 (2.0)		11 (1.5)
MMSE (mean ± SD)	28.6 (1.8)	28.6 (1.6)	0 (0.0)	28.6 (1.7)
MMSE			0 (0.0)	
<=26 (%)	36 (7.3)	20 (8.2)		680 (92.4)
>26 (%)	455 (92.7)	225 (91.8)		56 (7.6)
15-WLT				
Total words (mean ± SD)	28.5 (10.5)	29.1 (10.2)	3 (0.4)	28.7 (10.4)
Delayed recall (mean ± SD)	8.8 (3.3)	8.8 (3.4)	10 (1.4)	8.8 (3.3)
Rey-Osterrieth (mean ± SD)	19.0 (6.4)	20.4 (6.4)	10 (1.4)	19.4 (6.4)
DART vocabulary	48.5 (15.0)	49.2 (15.0)	9 (1.2)	48.7 (14.8)

### Cognitive function between groups

Those who showed abundant animal class knowledge (≥10) had a higher score on the animal fluency task (*B* = 5.64; 95% CI = [3.73; 7.56], *p* < 0.001). While not significant, the group with abundant animal class knowledge also showed on average a higher letter fluency score (*B* = 0.96; 95% CI = [−0.08; 2.00], *p* = 0.070). Moreover, the group who showed abundant animal class knowledge also had a higher total score on the 15-WLT (*B* = 2.73; 95% CI = [0.53; 4.93], *p* = 0.015) and delayed recall score (*B* = 1.01; 95% CI = [0.29; 1.73], *p* = 0.006) than those who did not. The groups scored similar on the Rey–Osterrieth Complex Figure (*B* = 0.53; 95% CI = [−0.84; 1.90], *p* = 0.449) and DART vocabulary score (*B* = −1.60; 95% CI = [−4.63; 1.43], *p* = 0.301). These results did not change after FDR correction.

Similarly in the sensitivity analysis (cut-off based on ≥7 animals), those with abundant animal class knowledge had a higher score on the animal fluency (*B* = 6.65; 95% CI = [5.44; 7.85], *p* < 0.001), letter fluency task (*B* = 1.13; 95% CI = [0.45; 1.82], *p* = 0.001), total score on the 15-WLT (*B* = 1.83; 95% CI = [0.37; 3.29], *p* = 0.014). While not significant, the group with abundant animal class knowledge showed a similar pattern as the main analysis of on average a higher delayed recall score on the 15-WLT (*B* = 0.46; 95% CI = [−0.02; 0.94], *p* = 0.060). In this analysis, those with abundant animal class knowledge also scored higher on the Rey–Osterrieth Complex Figure Test (*B* = 1.55; 95% CI = [0.65; 2.45], *p* < 0.001). The DART vocabulary score was similar between groups (*B* = −0.01; 95% CI = [−2.02; 2.00], *p* = 0.991). These results did not change after FDR correction.

## Discussion

We investigated the influence of abundant knowledge of animal class in semantic fluency performance on personal background factors and episodic memory performance. One-third of the sample could recall a series of 7 or more animals, and one-tenth could recall a series of 10 or more animals (with at most one interruption). Those who showed abundant animal class knowledge were older, more often male, and more often retired compared to those who did not. Moreover, showing abundant animal class knowledge was not only related to higher scores on the animal fluency task but also on letter fluency, and tasks of verbal and visual episodic memory. These results suggest that several personal background factors play a role in having abundant animal class knowledge that benefits animal fluency performance. However, the advantage is not only restricted to animal fluency and may reflect a higher level of memory functioning in general (both semantic and episodic memory), as well as better executive functioning, and thus does not seem to be disproportionally influencing animal fluency performance.

Our results showed that abundant animal class knowledge in the animal fluency task specifically applied to birds and fish. The specific recall of series of birds and fish might be influenced by the natural environment in the Netherlands. The nature reserves in the Netherlands mainly consist of water and forests, and the landscapes are flat. Regional and cultural influences can contribute to the animal class recalled on semantic fluency (Kempler et al., 1998; Eng et al., 2019). As such, the high population of birds and fish in the Netherlands may have influenced higher recall of these specific animals. With previous work showing the presence of qualitative differences in types of animal clusters across languages and cultures (e.g., in English vs. Mandarin speakers; Eng et al., 2019), future studies could focus on the characteristics of abundant animal class knowledge in animal fluency across cultures and nationalities, including differences in clustering and switching patterns. Moreover, individuals who produced animal series related to birds or fish classes might have had more outdoor or social activities in general; future studies should investigate this association.

Our results on several personal background factors in animal fluency are in line with previous literature that showed that male sex/gender and older age are related to being more involved in birdwatching and angling (Schroeder et al., 2006; Agahi and Parker, 2008; Moore et al., 2008; Conradie, 2015). In addition, our finding that those who show abundant animal class knowledge were more often retired is in line with previous research showing that leisure activities in early life are likely to be continued in later life, including during retirement (Peppers, 1976; Gibson and Singleton, 2012). In our study, abundant animal class knowledge was not associated with educational level or profession. Within the framework of cognitive reserve, education is commonly used as a proxy (Groot et al., 2018). However, the suitability of education as an appropriate proxy for cognitive reserve remains a subject of ongoing debate (Jones et al., 2011; Seblova et al., 2020; Avila et al., 2021). Other cognitive reserve factors related to personal background might maintain greater significance in determining an individual’s performance in semantic fluency.

We also found that those who showed abundant animal class knowledge have higher scores on several verbal and visual episodic memory functioning tasks. One concern around the validity of animal fluency performance is that a disproportional influence of personal background variables may mask cognitive impairment, in that the ability to still name many birds because of a bird-watching hobby remains even while being in the early stages of Alzheimer’s disease (Devita et al., 2020). Semantic loss is related to the preclinical phase of Alzheimer’s disease and mild cognitive impairment (Holtzer et al., 2020; Vonk et al., 2020). Previous research found that individuals with Alzheimer’s disease or at risk of Alzheimer’s disease recalled animals in smaller clusters (Troyer et al., 1998; Ford et al., 2020), with higher word typicality (Vita et al., 2014), and higher word frequency (Vonk et al., 2019). As such, individuals with abundant knowledge of a certain animal class who develop Alzheimer’s disease may decline in the number of words they can recall in a series because they are no longer able to access this knowledge. This hypothesis would fit with our finding that those with abundant animal class knowledge also show better performance on other cognitive tasks (episodic memory and executive functioning), such that the influence of personal background is not disproportionate to animal fluency alone. Our findings seem to validate the utility of the animal fluency task to measure cognitive impairment despite the benefit of personal background factors.

Strengths of our study include the large number of participants with item-level data on the animal fluency task and the small percentage of missing data on sample characteristics and variables of interest. While verbal fluency is usually administered within a one-minute time-frame, the animal fluency task in the SMART-MR study was administered during a time span of 2 min; nonetheless, the administration of verbal fluency tasks across 2 min is not uncommon either (Abrahams et al., 2000; Vonberg et al., 2014; Pereira et al., 2018). Although this alternative administration across 2 min may limit comparison of results with studies using a one-minute-design, the extended time frame may offer participants more opportunity to generate these long series within specific animal classes as time pressure is reduced. The time pressure in verbal fluency tasks often links to executive functioning abilities, and as such, a longer time span may allow for semantic processing abilities to prevail in this design (Pereira et al., 2018). Future studies could investigate the generation of abundant animal class knowledge in one-minute verbal fluency in comparison to the current study’s results. A limitation of our study is that there was no data on individuals’ hobbies or interests while it is likely that these additional personal background factors influence an individual’s recall pattern on the animal fluency task. In addition, the data available on profession was pre-defined in relatively broad profession categories that combined a variety of jobs, while specific jobs may provide more exposure to build abundant animal class knowledge than others. While episodic memory was assessed with three different tasks, offering a solid representation of this cognitive domain, another limitation of our study is that other domains of interest, i.e., executive function and semantic memory, were assessed with one test each. Therefore, it is not possible to know whether individuals with abundant animal class knowledge may have a better performance in other tasks of semantic memory, executive functioning, or other domains not covered by the study. Lastly, the population in this study consisted of a clinical sample of predominantly White and majority male middle-aged and older Dutch adults with a history of vascular disease; this sample distribution may impact the generalizability of the results to other populations.

This study addressed a validity concern often raised in item-level analyses of animal fluency performance that personal background factors may disproportionally influence overall performance and item-level metrics. Our findings showed that a relatively large percentage of Dutch individuals has abundant knowledge of specific animal classes, particularly birds and fish, and that this knowledge is influenced by several personal background factors. Regardless, those with abundant animal class knowledge also perform better on tasks of episodic memory and executive functioning. Therefore, the results suggest that the benefit of abundant animal class knowledge gained by personal background variables does not disproportionally influence animal fluency performance as individuals with such knowledge also performed better on other cognitive tasks unrelated to abundant knowledge of animal classes.

## Data availability statement

The original contributions presented in the study are included in the article/supplementary material, further inquiries can be directed to the corresponding author.

## Ethics statement

The studies involving humans were approved by the ethics committee of the UMC. The studies were conducted in accordance with the local legislation and institutional requirements. The participants provided their written informed consent to participate in this study.

## Author contributions

AS and JV contributed to conception and design of the study. AS performed the statistical analysis and wrote the first draft of the manuscript. MB and ET checked the analyses. All authors contributed to manuscript revision, read, and approved the submitted version.
